# Effects of *Pithecellobium Jiringa *Ethanol Extract against Ethanol-Induced Gastric Mucosal Injuries in *Sprague-Dawley *Rats

**DOI:** 10.3390/molecules17032796

**Published:** 2012-03-06

**Authors:** Ibrahim Abdel Aziz Ibrahim, Suhailah Wasmn Qader, Mahmood Ameen Abdulla, Amal R. Nimir, Siddig Ibrahim Abdelwahab, Fouad Hussain AL-Bayaty

**Affiliations:** 1 Department of Pharmacology, Faculty of Medicine, Universiti Teknology MARA, Shah Alam 40450, Malaysia; 2 Department of Biological Science, Faculty of Biosciences and Bioengineering, University of Technology Malaysia, UTM Skudai 81310, Johor, Malaysia; E-Mail: suhaylaqadir@yahoo.com; 3 Department of Molecular Medicine, Faculty of Medicine, University of Malaya, Kuala Lumpur 50603, Malaysia; E-Mail: mahmood955@yahoo.com; 4 Division of Basic Medical Sciences, Faculty of Medicine, Cyberjaya University College of Medical Sciences (CUCMS), Cyberjaya 63000, Selangor, Malaysia; E-Mail: aralmadi@yahoo.com; 5 Department of Pharmacy, Faculty of Medicine, University of Malaya, Kuala Lumpur 50603, Malaysia; E-Mail: siddigroa@yahoo.com; 6 Department of Restorative Dentistry, Faculty of Dentistry, Universiti Technology MARA, Shah Alam 40450, Malaysia; E-Mail: dribm74@gmail.com

**Keywords:** *Pithecellobium jiringa*, gastric ulcers, ethanol, omeprazole, histology

## Abstract

Current anti-gastric ulcer agents have side effects, despite the progression and expansion of advances in treatment. This study aimed to investigate the gastroprotective mechanisms of *Pithecellobium jiringa *ethanol extract against ethanol-induced gastric mucosal ulcers in rats. For this purpose, *Sprague* Dawley rats were randomly divided into five groups: Group 1 (normal control) rats were orally administered with vehicle (carboxymethyl cellulose), Group 2 (ulcer control) rats were also orally administered with vehicle. Group 3 (positive control) rats were orally administered with 20 mg/kg omeprazole, Groups 4 and 5 (experimental groups) received ethanol extract of *Pithecellobium jiringa *ethanol extract at a concentration of 250 and 500 mg/kg, respectively. Sixty minutes later, vehicle was given orally to the normal control group, and absolute ethanol was given orally to the ulcer control, positive control and experimental groups to generate gastric mucosal injury. The rats were sacrificed an hour later. The effect of oral administration of plant extract on ethanol-induced gastric mucosal injury was studied grossly and histology. The level of lipid peroxidation, (malondialdehyde—MDA), superoxide dismutase (SOD) and gastric wall mucus were measured from gastric mucosal homogenate. The ulcer control group exhibited severe gastric mucosal injury, and this finding was also confirmed by histology of gastric mucosa which showed severe damage to the gastric mucosa with edema and leucocyte infiltration of the submucosal layer. Pre-treatment with plant extract significantly reduced the formation of ethanol-induced gastric lesions, and gastric wall mucus was significantly preserved. The study also indicated a significant increase in SOD activity in gastric mucosal homogenate, whereas a significant decrease in MDA was observed. Acute toxicity tests did not show any signs of toxicity and mortality up to 5 g/kg. The ulcer protective effect of this plant may possibly be due to its preservation of gastric wall mucus along with increased SOD activity and reduction of oxidative stress (MDA)*. *The extract is non-toxic, even at relatively high concentrations.

## 1. Introduction

*Pithecellobium jiringa* (djengkol bean) is one of the members of the Leguminosae family. Its other scientific name is *Archidendron jiringa*. This plant originated from Southeast Asia, where it is known as jering in Malaysia, djengkol in Indonesian, krakos in Cambodia, and niang-yai in Thailand. In those countries, *P. jiringa* beans are typically consumed as a side dish with rice. Other people like to consume the beans with ground coconut [1]. Today *P. jiringa* is used in the production of organic pesticides. It contains djenkolic acid and sulphur which are able to inhibit and kill pests [2]. Significantly, the methanolic extract of *P. jiringa *was found to inhibit the activation of Epstein-Barr virus (EBV) by 30% or more. These findings highlight the potential of *P. jiringa* in inhibiting and targeting cancer cells [3]. In addition to containing proteins, dietary fibers and unsaturated fatty acids, it has been shown that *P. jiringa* contains protease inhibitors. As such, some of the inhibitory effects reported are for example the trypsin inhibitory activity which was found to be higher than the same activity described for soybeans [4]. This plant also found to contain high levels of polyphenolic compounds and has a potent antioxidant activity [5]. The present study was undertaken to investigate the gastroprotective mechanisms of *P. jiringa* ethanol extract against ethanol-induced gastric mucosal injury in experimental rats.

## 2. Results and Discussion

### 2.1. Acute Toxicity Study

**Figure 1:**
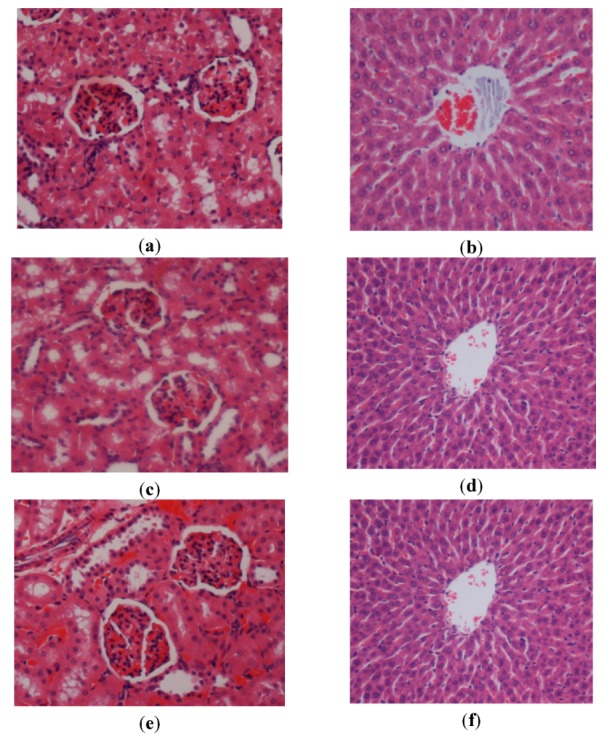
Histological sections of liver and kidney after the acute toxicity test. Rats (1a and 1b) treated with vehicle (CMC). Rats (1c and 1d) treated with 0.5 g/kg *P. jiringa* extract. Rats (1e and 1f) treated with 2 g/kg *P. jiringa* extract. There is no significant differences in structures of liver and kidney between treated and control groups (H & E stain 20×).

In the acute toxicity study animals were treated with the *P. jiringa* extract at a dose of 0.5 g/kg and 2 g/kg and kept under observation for 14 days. All the animals remained alive and did not manifest any significant visible of toxicity at these doses. Thus, clinical observations, serum biochemistry, and histopathology data did not show any significant differences between control and treated groups (Figure 1, Table 1 and Table 2). We conclude that *P. jiringa* extract orally administered to rats was safe and that no drug-related toxicity was detected, even at the highest dose investigated.

**Table 1:** Renal function test of rats in acute toxicity study of *P. jiringa* extract.

Dose (5 mL/kg)	Sodium (mmol/L)	Pottasium (mmol/L)	Chloride (mmol/L)	CO_2 _(mmol/L)	Anion gap (mmol/L)	Urea (mmol/L)	Creatinine (µmol/L)
Vehicle (CMC)	137.25 ± 0.41	5.03 ± 0.17	102.03 ± 0.15	23.03 ± 0.82	18.16 ± 0.72	5.63 ± 0.41	50.18 ± 1.34
LD (0.5 g/kg)	137.11 ± 0.42	5.21 ± 0.15	103.61 ± 1.22	21.74 ± 0.17	17.07 ± 1.35	4.96 ± 0.43	48.97 ± 0.81
HD (2 g/kg)	137.21 ± 0.50	5.12 ± 0.14	103.07 ± 0.76	22.8 ± 0.86	17.73 ± 0.51	5.93 ± 0.39	48.60 ± 1.80

Values expressed as mean ± S.E.M. There are no significant differences between groups. Significant value at *p <* 0.05.

**Table 2:** Liver function test of rats in acute toxicity study of *P. jiringa* extract.

Dose (5 mL/kg)	Total protein (g/L)	Albumin (g/L)	Globulin (g/L)	TB (µmol/L)	CB (µmol/L)	AP(IU/L)	ALT(IU/L)	AST(IU/L)	GGT(IU/L)
Vehicle (CMC)	72.33 ± 1.44	11.39 ± 0.51	59.09 ± 1.37	1.96 ± 0.19	0.96 ± 0.16	134.78 ± 9.51	53.05 ± 3.24	154.61 ± 9.37	4.96 ± 0.93
LD (0.5 g/kg)	71.41 ± 0.52	11.65 ± 0.33	59.45 ± 0.38	2.12 ± 0.15	1.00 ± 0.00	133.37 ± 8.66	51.90 ± 1.37	156.07 ± 3.66	5.03 ± 1.28
HD (2 g/kg)	71.84 ± 1.03	11.62 ± 0.14	60.11 ± 0.68	1.84 ± 0.24	1.00 ± 0.00	134.13 ± 6.55	52.21 ± 3.22	155.02 ± 5.38	5.38 ± 1.09

Values expressed as mean ± S.E.M. There are no significant differences between groups. *p* *<* 0.05 were considered statistically significant. TB: Total bilirubin; CB: Conjugated bilirubin; AP: Alkaline phosphatase; ALT: Alanine aminotransferase; AST: Aspartate aminotransferase; GGT: G-Glutamyl Transferase.

### 2.2. Gross Appearance of Gastric Lesions

**Figure 2:**
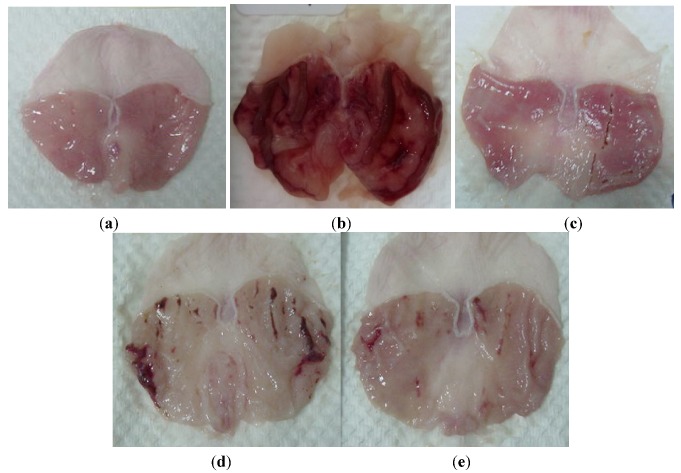
Gross appearance of the gastric mucosa in rats. (**a**) Rats pre-treated with CMC (normal control). No disturbance of gastric mucosa. (**b**) Rats pre-treated with CMC (ulcer control). Severe injuries are seen in the gastric mucosa. Absolute ethanol produced extensive visible hemorrhagic necrosis of gastric mucosa. (**c**) Rats pre-treated with of omeprazole (20 mg/kg). Injuries to the gastric mucosa are much milder compared to the injuries seen in the ulcer control rats. (**d**) Rats pre-treated with *P.* *jiringa* (250 mg/kg). Moderate injuries to the gastric mucosa are seen. (**e**) Rat pre-treated with *P.* *jiringa* (500 mg/kg). Mild injuries are seen in the gastric mucosa; and flattening of the gastric mucosa is seen.

Rats pretreated with omeprazole (GIII), or with both doses of ethanol extract (GIV and GV) before being given absolute alcohol showed significantly reduced areas of gastric ulcer formation compared to GII (Figure 2, Table 3). Flattening of gastric mucosal folds was also observed in GIV and GV rats. Moreover, the mucosal damage was significantly reduced in size and severity in GIII, GIV and GV. On the other hand, GII animals showed sever ulcer formation (Figure 2, Table 3).

**Table 3:** Effect of *P. jiringa* on ethanol-induced changes in gastric wall mucus, Superoxide dismutase, Lipid peroxidation and ulcers areain rats.

Group	Pre-treatment (5 mL/kg)	Post-treatment (5 mL/kg)	Gastric wall mucus (µg Alcian blue/g wet stomach)	SOD (mU of SOD/mg tissue)	MDA (μmol of MDA/mg tissue)	ulcers area (mm^2^) Mean ± SEM	Inhibition (%)
GI	CMC	CMC	930.50 + 27.44 ^a^	165.35 ± 7.68 ^a^	30.24 ± 4.09 ^a^	-	-
GII	CMC	Absolute ethanol	562.21 + 21.33 ^b^	75.15 ± 4.29 ^b^	91.67 ± 6.12 ^b^	836.67 ± 2.06 ^a^	-
GIII	20 mg/kgOmeprazole	Absolute ethanol	815.07 + 25.17 ^c^	135.18 ± 5.94 ^a^	44.06 ± 4.88 ^c^	68.33 ± 2.05 ^b^	92.01
GIV	250 mg/kg *P.* *jiringa*	Absolute ethanol	795.67 + 19.98 ^c^	152.33 ± 5.79 ^a^	47.17 ± 3.97 ^c^	228.17 ± 1.51^c^	72.17
GV	500 mg/kg *P.* *jiringa*	Absolute ethanol	857.26 + 26.03 ^c^	162.06 ± 6.17 ^a^	40.08 ± 4.36 ^c^	156.33 ± 1.84 ^d^	80.55

All values are expressed as mean ± standard error mean. Means with different superscripts are significantly different. The mean difference is significant at the *p <* 0.05 level.

### 2.3. Histological Changes of Gastric Mucosa

**Figure 3:**
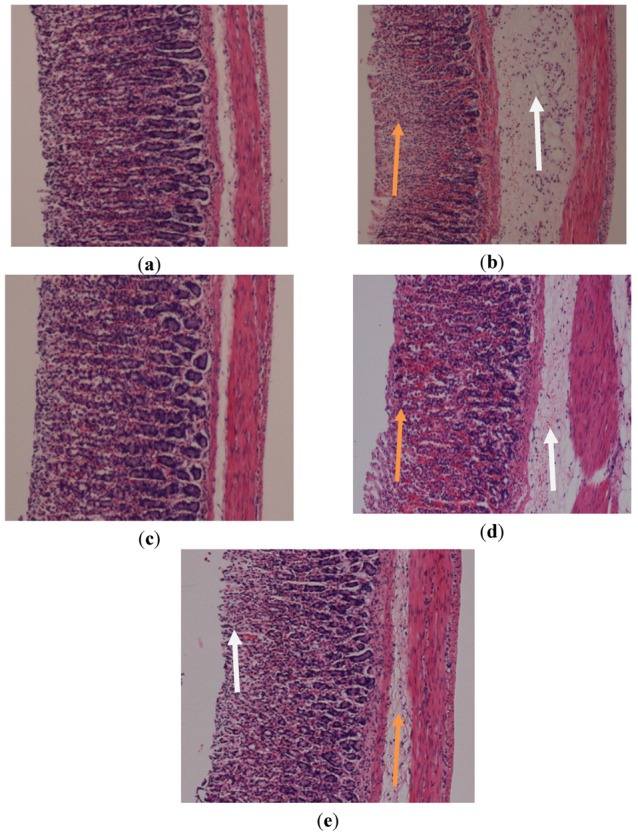
Histological study of the absolute ethanol-induced gastric mucosal damage in rats. (**a**) Rats pre-treated with CMC (normal control). No disturbance of gastric mucosa. (**b**) Rats pre-treated with CMC (ulcer control). There is severe disruption to the surface epithelium and necrotic lesions penetrate deeply into mucosa (orange arrow) and extensive edema of submucosal layer and leucocytes infiltration are present (white arrow). (**c**) Rats pre-treated with omeprazole (20 mg/kg). Very mild disruption of the surface epithelium mucosa is present, but deep mucosal damage is absent. (**d**) Rats pre-treated with *P.* *jiringa* (250 mg/kg). There is moderate disruption to the surface epithelium (orange arrow) with edema and leucocyte infiltration of the submucosal layer (white arrow). (**e**) Rat pre-treated with *P.* *jiringa* (500 mg/kg). Mild disruptions of surface epithelium are present but deep mucosal damage is absent (orange arrow). There is slight edema with leucocyte infiltration of the submucosal layer (white arrow) (H&E stain 10×).

Microscopic observations of ethanol-induced gastric lesions in ulcers control rats (GII) illustrated significant and extensive damage in the gastric mucosa with oedema and inflammatory cells infiltration in the submucosal layer (Figure 3). Rats in GIV and GV and those treated with omeprazole (GIII), displayed better protection of their gastric mucosa as seen by the reduction of the ulcerated areas (Figure 3). Reduced submucosal oedema and the inflammatory reactions were also observed in these groups. The histological appearance in GIV was somewhat similar to GIII(Figure 3).

### 2.4. Effect of *P. jiringa* on Ethanol-Induced Changes in the Gastric Wall Mucus

The treatment of rats with ethanol significantly decreased the Alcian blue binding capacity of the gastric wall mucus in the ulcer control animals. Pre-treatment with *P. jiringa* extract at doses of 250 and 500 mg/kg significantly enhanced the Alcian blue binding capacity of the gastric mucosa and increased the amount of gastric mucus in ethanol-ulcerated rats (Table 3).

### 2.5. Effect of *P. jiringa* on Ethanol-Induced Changes in Gastric Antioxidant Enzyme (SOD)

Ethanol reduced the SOD activity in ulcer control stomachs pretreated with vehicle when compared with normal group. Pre-treatment with plant extract increased the activity of the enzyme compared with the ulcer control group. Similarly, omeprazole increased SOD activity compared with the ulcer control group (Table 3).

### 2.6. Measurement of Membrane Lipid Peroxidation (μmol MDA/mg of Tissue)

Lipoperoxidation in the ulcer control stomachs was significantly higher when compared with the normal control group. In the stomachs of the animals pretreated with *P. jiringa* or omeprazole a decrease in lipoperoxidation was observed compared with the ulcer control stomach (Table 3). The acute toxicity test did not show any signs of toxicity or mortality when administered orally up to 2 g/kg. Peptic ulcers are caused by an imbalance between the protective and aggressive mechanisms of the mucosa and are the result of the association of several endogenous factors and aggressive exogenous factors that are related to living conditions [6]. Ethanol produces necrotic lesions by direct necrotizing action, which in turn reduces defensive factors, including the secretion of bicarbonate and production of mucus [7]. The gastric wall mucus is thought to play an important role as a defensive barrier against gastrointestinal damage [8]. Pre-treated with the *P. jiringa* extract significantly increased the gastric mucus content in rats with ethanol-induced ulcers suggests that the gastroprotective effect of this plant is mediated partly by preservation of the gastric wall mucus. Gastric wall mucus depletion induced by ethanol is also one of the pathogenic mechanisms responsible for gastric lesions [9]. It has been reported that certain anti-ulcer drugs increase the amount of gastric mucus secretion in the gastric mucosa [10]. The increase in bound Alcian blue indicates the protective effect of orally administered *P. jiringa*, which may occur through the formation of protective complexes between this plant extract and the mucus that acts as a barrier against necrotizing agents introduced in the stomach [11]. The preservation of adherent mucus on the glandular mucosa is one of the contributing factors in the prevention of gastric mucosal damage induced by chemical irritants [12]. 

The oral administration of absolute ethanol to rats produces linear hemorrhagic lesions, extensive submucosal edema, inflammatory cell infiltration, and epithelial cell loss in the stomach, which are the typical characteristics of alcohol injury [13]. The pathogenesis of ethanol-induced gastric mucosal damage occurs directly and indirectly through various mediators, such as lipoxygenase, cytokines, and oxygen-derived free radicals [14]. Mucus secretion is regarded as a crucial defensive factor in the protection of the gastric mucosa from gastric lesions [15]. The results of the present study showed that *P. jiringa* extract has an effective anti-ulcer activity against ethanol-induced gastric mucosal injury. The plant extract increased the mucus of the gastric wall, which is consistent with results reported by Thirunavukkarasu *et al.* [16]. Omeprazole, a proton pump inhibitor (PPI), exhibits an anti-secretory and protective effect [17]. Omeprazole is effective in treating peptic ulcer disease and gastroesophageal reflux in short- and long-term use [18]. The pathogenesis of mucosal damage in the stomach includes the generation of reactive oxygen species (ROS) that appear to play a vital role in the formation of lipid peroxides and is accompanied by the impairment of the activity of antioxidant enzymes in cells [19].

Oxidative stress plays an important role in the pathogenesis of various diseases, including gastric ulcer disease, with antioxidants reported to play a significant role in the protection of the gastric mucosa against various necrotic agents [20]. Antioxidants could help protect the cells from damage caused by oxidative stress and enhance the body’s defense systems against degenerative diseases. The administration of antioxidants inhibits ethanol-induced gastric injury in rats [21]. *P. jiringa* extract has been shown to contain antioxidants [5], and it is likely that the gastroprotective effect exerted by this plant extract could be attributed to its antioxidant properties. Our results revealed the protection of the gastric mucosa and inhibition of leukocyte infiltration to the gastric wall in rats pretreated with *P. jiringa* extract. The activation and infiltration of neutrophils appear to be involved in the initial processes that form the lesions. Similarly, Abdulla *et al. * [22] demonstrated that the reduction of neutrophil infiltration into ulcerated gastric tissues promoted the prevention of gastric ulcers in rats. Wasman *et al.* [23] showed that the oral administration of plant extract before ethanol administration significantly decreased neutrophil infiltration into the gastric mucosa. Neutrophils mediate lipid peroxidation through the production of superoxide anions [24]. Neutrophils are a major source of inflammatory mediators and can release potent reactive oxygen species, such as superoxide, hydrogen peroxide and myeloperoxidase-derived oxidants. These reactive oxygen species are highly cytotoxic and can induce tissue damage [25]. We observed a flattening of the mucosal folds, which suggests that the gastroprotective effect of the *P. jiringa* leaf extract might be due to a decrease in gastric motility. It has been reported that changes in gastric motility may play a role in the development and prevention of experimental gastric lesions [22]. The relaxation of the circular muscles may protect the gastric mucosa through the flattening of the folds. This flattening will increase the mucosal area exposed to necrotizing agents and reduce the volume of the gastric irritants on the rugal crest [22,23].

MDA is the final product of lipid peroxidation and is used to determine lipid peroxidation levels [26]. Gastric MDA was increased in the ulcer control group and decreased by *P. jiringa* extract administration, another indicator of the potential antioxidant functionality of this plant. Lipid peroxidation causes a loss in membrane fluidity, impaired ion transport, membrane integrity, and finally, the loss of cellular functions.

The gastric tissue homogenate from animals pre-treatment with omeprazole or plant extract showed significant decrease in the levels of MDA and elevation of the levels of SOD in response to absolute ethanol. Free radicals and reactive oxygen species (ROS) are continuously produced in the human body. These oxygen species are a cause of cellular damage. SOD converts superoxide to hydrogen peroxide, which is transformed into water by catalase in lysosomes or by glutathione peroxidase in the mitochondria [27].

SOD, which plays an important role in protecting gastrointestinal mucosa, owes its antioxidant properties to its ability to scavenge superoxide anions. The increase in SOD level in the experimental groups of the present study clearly point to an antioxidant mechanism underlying its gastroprotective action, while the ability of the plant extract to prevent lipid peroxidation *in vitro* reinforces its potential use as a therapeutic drug for free radical pathologies. Our results revealed that the activity of SOD was reduced in gastric tissue homogenates from the ulcer control group. The decrease in the activity of SOD in tissue homogenate may be due to the increased production of reactive oxygen radicals, which can reduce the activity of this enzyme [28]. The reduction of SOD enzyme in gastric homogenate may lead to a number of deleterious effects. Lipid peroxidation has been observed to be an important pathophysiological event in a variety of diseases, including gastric ulcer disease [29]. Lipinski [30] showed that reactive oxygen species can induce cellular events such as enzyme inactivation, DNA strand cleavage and membrane lipid peroxidation. Oxidative stress is associated with the peroxidation of cellular lipids [28]. In the ulcer control rats, the MDA levels were observed to be higher than the normal control rats, indicating lipid peroxidation. The levels of MDA in the gastric tissue of rats that received *P. jiringa* were significantly decreased when compared to the ulcer control rats. This result suggests that the plant extract could improve the pathological condition of gastric ulcer disease by a reduction of lipid peroxidation.

## 3. Experimental

### 3.1. Drugs and Chemicals

In this study, Omeprazole was used as the reference anti-ulcer drug, and was obtained from the University Malaya Medical Centre (UMMC) Pharmacy. The drug was dissolved in 0.5% carboxymethylcellulose and given orally to six rats (GII) in concentrations of 20 mg/kg 5 mL/kg) according to the recommendations of Mahmood *et al*. [31].

### 3.2. Preparation of Carboxymethylcellulose (Vehicle)

0.5% carboxymethylcellulose (CMC) was prepared by adding 0.5 g of absolute carboxylmethyl cellulose to 100 mL of distilled water.

### 3.3. Preparation of the Ethanol Extraction

The plant was peeled and beans were ground. Ground beans (100 g) were weighed and mixed with 95% ethanol (1,000 mL) (*i.e*., 1:10 ratio) [31]. Then, the mixture was placed in a conical flask which was then covered with aluminum foil. The mixture was kept at room temperature 7 days for maceration. Next, the extract was filtered and concentrated using an EYELA rotator evaporator. Once evaporation was concluded, the extract was collected in a universal bottle and kept in incubator at 45 °C overnight. The dry extract was then dissolved in CMC and administered orally to rats in concentrations of 250 and 500 mg/kg body weight (5 mL/kg body weight) according to the recommendation of Wasman *et al*. [23].

### 3.4. Acute Toxicity Test and Experimental Animals

Adult healthy male and female Sprague-Dawley rats (6–8 weeks old) were obtained from the Animal House, Faculty of Medicine, University of Malaya, Kuala Lumpur (Ethic Permit No. PM/27/07/2010/MAA (R). The rats weighed between 150–180 g. The animals were given standard rat pellets and tap water. The acute toxicity study was used to determine a safe dose for the extract. Thirty six Sprague Dawley rats (18 males and 18 females) were assigned equally each into three groups labeled as vehicle (CMC); 0.5 g/kg and 2 g/kg of *P. jiringa* extract preparation, respectively. The animals were fasted overnight (food but not water) prior to dosing. Food was withheld for a further 3 to 4 h after dosing. The animals were observed for 30 min and 2, 4, 8, 24 and 48 h after the administration for the onset of clinical or toxicological symptoms. Mortality, if any was observed over a period of 2 weeks. The animals were sacrificed on the 15th day. Histology, hematological and serum biochemical parameters were determined following standard methods [23,31]. The study was appproved by the Ethics Committee for Animal Experimentation, Faculty of Medicine, University of Malaya, Malaysia. Throughout the experiments, all animals received human care according to the criteria outlined in the “*Guide for the Care and Use of Laboratory Animals*” prepared by the National Academy of Sciences and published by the National Institutes of Health.

### 3.5. Experimental Animals for Gastric Ulcer

Adult male Sprague-Dawley rats (200–250 g) were provided by the Animal House, Faculty of Medicine, University of Malaya (Ethics Permit No. PM/27/07/2010/MAA (R). All animals used for the study had ethical clearance from the Animal User’s Committee of the Faculty of Medicine, University of Malaya. The rats were deprived of food for 24 h before the experiment and only allowed access to water 2 h prior to the experiment. They were randomly divided into five groups, six rats per group, and individually housed in cages with a wide-mesh wire bottom to prevent coprophagy and dominancy. Throughout the experiment, all animals received human care according to the criteria outlined in the “*Guide for the Care and Use of Laboratory Animals*”.

### 3.6. Treatment

Gastric ulcers were induced by orogastric intubation of absolute ethanol (5 mL/kg) according to the method described by Abdulla *et al.* [22] (Table 3). The rats were euthanized 60 minutes later (under an overdose of xylazin and ketamine anesthesia) and their stomachs were immediately excised (Wasman *et al.* [23]).

### 3.7. Gross Gastric Lesions Evaluation

Ulcers of the gastric mucosa appear as elongated bands of hemorrhagic lesions parallel to the long axis of the stomach. Gastric mucosa of each rat was thus examined for damage. The length and width of the ulcer (mm) were measured by a planimeter (10 × 10 mm^2^ = ulcer area) under dissecting microscope (1.8 ×). The ulcerated area was measured by counting the number of small squares, 2 mm × 2 mm, covering the length and width of each ulcer band. The sum of the areas of all lesions for each stomach was applied in the calculation of the ulcer area (UA) where in the sum of small squares × 4 × 1.8 = UA (mm^2^) according to the recommendation of Mahmood *et al*. [31]. The inhibition percentage (I.0%) was calculated by the following formula according to the recommendation of Wasman *et al. *[23]:

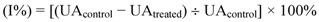


### 3.8. Determination of Gastric Wall Mucus

Gastric wall mucus was determined according to the modified procedure of Corne *et al*. [32]. The glandular segment of the stomach was separated from the lumen of the stomachs, weighed and transferred immediately to 10 mL of 0.1% w/v Alcian blue solution (in 0.16 mol sucrose solution buffered with 0.5 mL sodium acetate at pH 5). Tissue was stained for 2 h in Alcian blue and excess dye was removed by two successive rinses with 10 mL of 0.25 M sucrose. Dye complexed with the gastric wall mucus was extracted with 10 mL of 0.5 mol magnesium chloride, which was intermittently shaken for 1 min at 30 min intervals for 2 h. Four milliliters of blue extract were then vigorously shaken with an equal volume of diethyl ether. The resulting emulsion was centrifuged at 3,000 g for 10 min and the absorbance of the aqueous layer was recorded at 580 nm. The quantity of Alcian blue extracted per gram of wet glandular tissue was then calculated.

### 3.9. Microscopic Studies

The opened stomachs were preserved in 10% buffered formalin overnight after cutting them into small pieces. The tissues were processed by automated tissue processing the next day. Next, the biopsies were embedded in paraffin wax and sectioned at 5 µm thickness by microtome and then stained with haematoxylin and esin (H & E) [22,33]. The tissue sections were assessed for histopathological changes such as congestion, edema, hemorrhage and necrosis using a light microscope.

### 3.10. Estimation of SOD Antioxidant Enzyme and Proteins Concentration

Superoxide dismutase (SOD) activity was determined according to the method described by Marklund and Marklund [34]. The reaction mixture consisted of 0.5 mL of Tris-buffer (50 mM; pH-8.2), 0.5 mL pyragallol (0.5 mmol), 0.5 mL EDTA (1 mmol), and in different volumes, 0.025 mL, 0.05 mL, 0.075 mL, and 0.1 mL of tissue homogenate. The change in absorbance was recorded at 420 nm. Activity was reported by its ability to inhibit 50% reduction of pyragallol and the result is expressed as mU/min/mg protein.

### 3.11. Measurement of the Membrane Lipids Peroxidation

The rate of lipoperoxidation in the gastric mucous membrane was estimated by determination of malondialdehyde (MDA) using the Thiobarbituric Acid Reactive Substances (TBARS) test. The stomachs were washed with saline to minimize the interference of hemoglobin with free radicals and to remove blood adhered to the mucous membrane. The stomachs were homogenized to 10% of tissue with potassium phosphate buffer. Then, 250 μL was removed and stored at 37 °C for 1 h, after which 400 μL of 35% perchloric acid was added, and the mixture was centrifuged at 14,000 rpm for 20 min at 4 °C. The supernatant was removed, mixed with 400 μL of 0.6% thiobarbituric acid and incubated at 95–100 °C for 1 h. After cooling, the absorbance at 532 nm was measured. A standard curve was generated using 1,1,3,3-tetrametoxypropane. The results were expressed as nmol of MDA/mg of protein. The concentration of proteins was measured using the method described by Bradford [35]. Measurement of total protein in the stomach sample after ethanol-induced lesions. The method is based on the interaction of the Coomassie Blue G250 dye with proteins. At the pH of the reaction, the interaction between proteins of high molecular weight and the dye causes a shift in the dye to the anionic form, which absorbs strongly at 595 nm. Solutions of albumin standard, distilled water, buffer and samples were added to the wells. For sample preparation, 2 μL of sample and 38 μL of buffer were added to each well. Then, 200 μL Bradford’s solution (diluted 5×) was added to each well. After 5 min, a reading was taken at the wavelength of 595 nm [35].

### 3.12. Statistical Analysis

All values were reported as mean ± S.E.M. The statistical significance of differences between groups was assessed using one-way ANOVA. A value of *p* *<* 0.05 was considered significant.

## 4. Conclusions

We conclude that *P. jiringa* plays a protective role against gastric ulcers, and its anti-ulcer effect is related to an increase of adherent mucus, which may inhibit the generation of oxygen-derived free radicals. The gastroprotective effects of this plant in experimental ulcers induced by absolute ethanol could be related to its SOD activity and a decrease in lipid peroxidation.
